# 

**Published:** 1885-01

**Authors:** 


SURG GENLS OFFS

Vol. XX. January, 1885. No. 4.

### The Bistoury.

Thad S. Up de Graff, M.D.
EDITOR.

ELMIRA, N.Y.

